# Microbial hauberks: composition and function of surface layer proteins in gammaproteobacterial methanotrophs

**DOI:** 10.1128/aem.01364-24

**Published:** 2024-12-31

**Authors:** Richard Hamilton, William Gebbie, Chynna Bowman, Alex Mantanona, Marina G. Kalyuzhnaya

**Affiliations:** 1 Biology Department, San Diego State University 7117 , San Diego, California, USA; University of Nebraska-Lincoln, Lincoln, Nebraska, USA

**Keywords:** methanotrophy, surface (S-)layer, glycoprotein, *Methylotuvimicrobium*, Methylomicrobium

## Abstract

Many species of proteobacterial methane-consuming bacteria (methanotrophs) form a hauberk-like envelope represented by a surface (S-) layer protein (SLP) matrix. While several proteins were predicted to be associated with the cell surface, the composition and function of the hauberk matrix remained elusive. Here, we report the identification of the genes encoding the hauberk-forming proteins in two gamma-proteobacterial (Type I) methanotrophs, *Methylotuvimicrobium buryatense* 5GB1 (EQU24_15540) and *Methylotuvimicrobium alcaliphilum* 20Z^R^ (MEALZ_0971 and MEALZ_0972). The proteins share 40% amino acid (AA) sequence identity with each other and are distantly related to the RsaA proteins from *Caulobacter crescentus* (20% AA sequence identity). Deletion of these genes resulted in loss of the characteristic hauberk pattern on the cell surface. A set of transcriptional fusions between the MEALZ_0971 and a superfolder green fluorescent protein (sfGFP) further confirmed its surface localization. The functional roles of the hauberk and cell-surface-associated proteins, including MEALZ_0971, MEALZ_0972, EQU24_15540, and a copper-induced CorA protein, were further investigated via gene expression studies and phenotypic tests. The hauberk core protein of *M. alcaliphilum* 20Z^R^ showed constitutive expression across 18 growth conditions with reduced growth at high salinity, high methanol, and metal-limited conditions, suggesting a role in cell-envelope stability and metal scavenging. Overall, understanding the genetics, composition, and cellular functions of S-layers contributes to our knowledge of methanotroph adaptation to environmental perturbations and opens a promising prospect for (nano)biotechnology applications.

**IMPORTANCE:**

Understanding the genetics, composition, and cellular functions of the cell envelope proteins, such as S-layers, contributes to our knowledge of microbial cell biology and stress responses and molecular adaptations to environmental perturbations. In addition, this study opens a promising prospect for (nano)biotechnology applications of methane-derived self-assembling protein materials.

## INTRODUCTION

Surface (S-) layers that consist of proteins or glycoproteins forming lattices covering the cells (i.e., SLPs) have been identified in many prokaryotic organisms spanning both bacteria and archaea (1–3). These proteins are abundant proteins synthesized by organisms at a high metabolic cost and, thus, scientists have wanted to characterize their functions and determine their role in specific environments (3). The predicted functions of SLPs in different bacterial species vary based on whether the organisms are Gram-positive or Gram-negative. Predicted functions include cell adhesion, protection, virulence factors, anchoring sites, receptors for phages, porin sites, and molecular/ion traps (1). SLPs are unique self-assembling cellular structures with numerous applications in (nano)biotechnology as well as materials and environmental sciences (4). One of the most characterized SLP in Gram-negative bacteria is from *Caulobacter crescentus*. Its S-layer protein was identified to be a 98-kDa protein (RsaA) that is transported to the outer membrane through a type 1 secretion system (T1SS) (5, 6). The RsaA protein forms a hexamer (p6 symmetry) anchoring to the outer membrane by liposaccharides and makes up about 30% of total cell protein (7–9).

S-layer protein lattices have been identified in numerous methane-consuming bacteria (methanotrophs), including alpha and gammaproteobacteria (10–13). Their S-layers have been shown to form matrixes with p6, p4, or p2 symmetry (10–14). In *Methylococcus capsulatus*, for example, S-layers are arranged in tetragonal (p4) symmetry (15). In the haloalkaliphilic *Methylotuvimicrobium* strains, which are gammaproteobacterial methanotrophs, the hauberk-like S-layer envelope has cup-shaped structures packed in hexagonal (p6) symmetry (13, 16).

It has been suggested that the methanotroph hauberk-like envelope is formed by glycosylated proteins; however, the genetics have remained elusive (13). Studies that determine dominant outer cell envelope proteins for a haloalkaliphilic *Methylotuvimicrobium* culture (*M. alcaliphilum* 20Z) have revealed a number of proteins including CorA and CorB homologs (17). The CorA protein has been implicated in copper acquisition in some methanotrophs (17–19). In *M. album* BG8, a *corA* mutant showed impaired growth on methane (18, 20). A similar phenotype was observed for *M. alcaliphilum* 20Z^R^. The *M. alcaliphilum* 20Z^R^ ∆*corA* (MEALZ_2831) mutant was unable to grow on methane but grew on methanol, while the *corB* (MEALZ_2832) mutant grew faster on methane compared to the wild-type (17). In addition, it has been shown that the *corA* mutant showed major ultrastructural changes and appeared to contain the S-layer protein within the intercellular space and only loosely attached to the outer cell surface. Thus, it has been suggested that CorA is involved not only in copper acquisition but also in attaching the S-layer to the outer cell envelope (17). A highly expressed cell envelope protein (WP_009059494) has recently been characterized in an acidophilic verrucomicrobial methanotrophic strain *Methylacidiphilum fumariolicum* SolV (21). The PRED-TMBB and AlphaFold2 simulations predicted that the protein forms a porin with ten antiparallel β-barrels. The immunogold and negative staining transmission electron microscopy of the SolV cells and purified protein preparations confirmed that the WP_009059494 is localized on the cell surface, but it does assemble into regular lattices (21). Considering that S-layer lattices and outer membrane proteins (OMP proteins) have mostly been investigated in extremophilic methanotrophs, their functions are often associated with envelope stability.

In this study, we focus on the identification of genes that encode S-layer proteins in *M. alcaliphilum* 20Z^R^ and *M. buryatense* 5GB1. Both *Methylotuvimicrobium* strains have been shown to possess hauberk-like S-layers with hexagonal cup-like structures. We investigated the composition of their cell envelope proteins, particularly the matrix core protein, and explored S-layer protein roles in cell envelope stability, metal uptake, and methanotrophic metabolism by integrating -omics studies, mutagenesis, and cell-surface imaging. This study solves the decade-long puzzle of the hauberk-protein composition and the role of the surface matrix in gammaproteobacterial methanotrophs, while also paving the way to employ the hauberk matrix as functionalized nanomaterials (4).

## RESULTS

### Identification of putative S-layer proteins (SLPs): -omics studies

Initial SLP candidates were identified using existing RNAseq transcriptomics data sets. Reasoning that the SLP must be generated in high amounts and noting that most SLPs are larger than average proteins, the top 50 expressed genes in existing data sets of methane *M. buryatense* 5GB1 and *M. alcaliphilum* 20Z^R^ were sorted based on size (22). One candidate (MEALZ_0971) in *M. alcaliphilum* 20Z^R^ and its homolog (EQU24_15540) in *M. buryatense* 5GB1 were identified as both highly expressed and greater than 5 kb in size in both methanotrophs. The proteins share 40% amino acid (AA) sequence identity. A smaller gene (MEALZ_0972) adjacent to MEALZ_0971 was identified in *M. alcaliphilum* 20Z^R^. Based on transcript mapping data, MEALZ_0972 forms an operon with MEALZ_0971. The small protein does not have a homolog in the genome of *M. buryatense* 5GB1.

**Fig 1:**
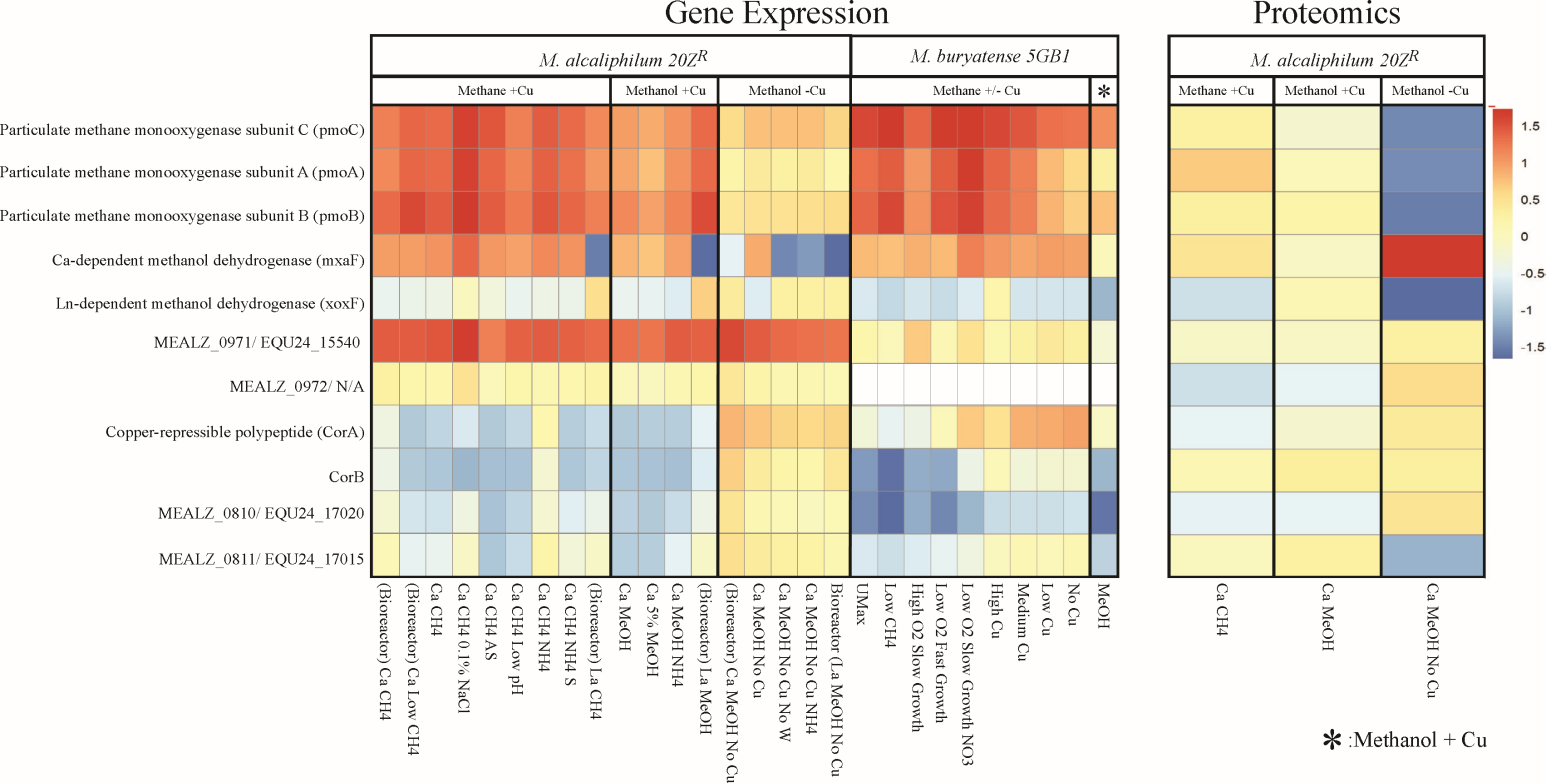
Heatmap of transcriptomic and proteomic expression levels in the putative outer membrane protein in *M. alcaliphilum 20Z^R^* and *M. buryatense* 5GB1genes compared to key metabolic enzymes across different growth conditions.

We compared the expression levels of MEALZ_0971 and MEALZ_0972 with those of the most abundant cellular proteins, such as particulate methane monooxygenase (pMMO) and the methanol dehydrogenases (MxaF and XoxF) (Fig. 1). Transcriptomic data sets for *M. alcaliphilum* 20Z^R^ were analyzed across 18 different environmental conditions that are known to control gene expression levels including +/-copper, calcium vs lanthanides, methanol vs methane, and high to low salinities. Two bioreactor cultures of *M. buryatense* 5GB1 were also evaluated (methane and methanol) (23–25). A set of proteins previously identified as associated with the hauberk envelope were included, including CorA, CorB, MEALZ_0810, and MEALZ_0811 (Fig. 1). Only the large proteins (MEALZ_0971 and EQU24_15540) have significantly above-average gene expression in both strains. In M. *alcaliphilum* 20Z^R^, the MEALZ_0971 gene product also showed the highest levels of spectral counts in the proteome. The expression of CorA and MEALZ_0810- MEALZ_0811 correlated with copper supplementation, with all genes being induced by copper limitation. The expression of the MEALZ_0972 gene was constitutive across most conditions, but significantly lower than the MEALZ_0971 expression.

### Comparative genomics

**Fig 2:**
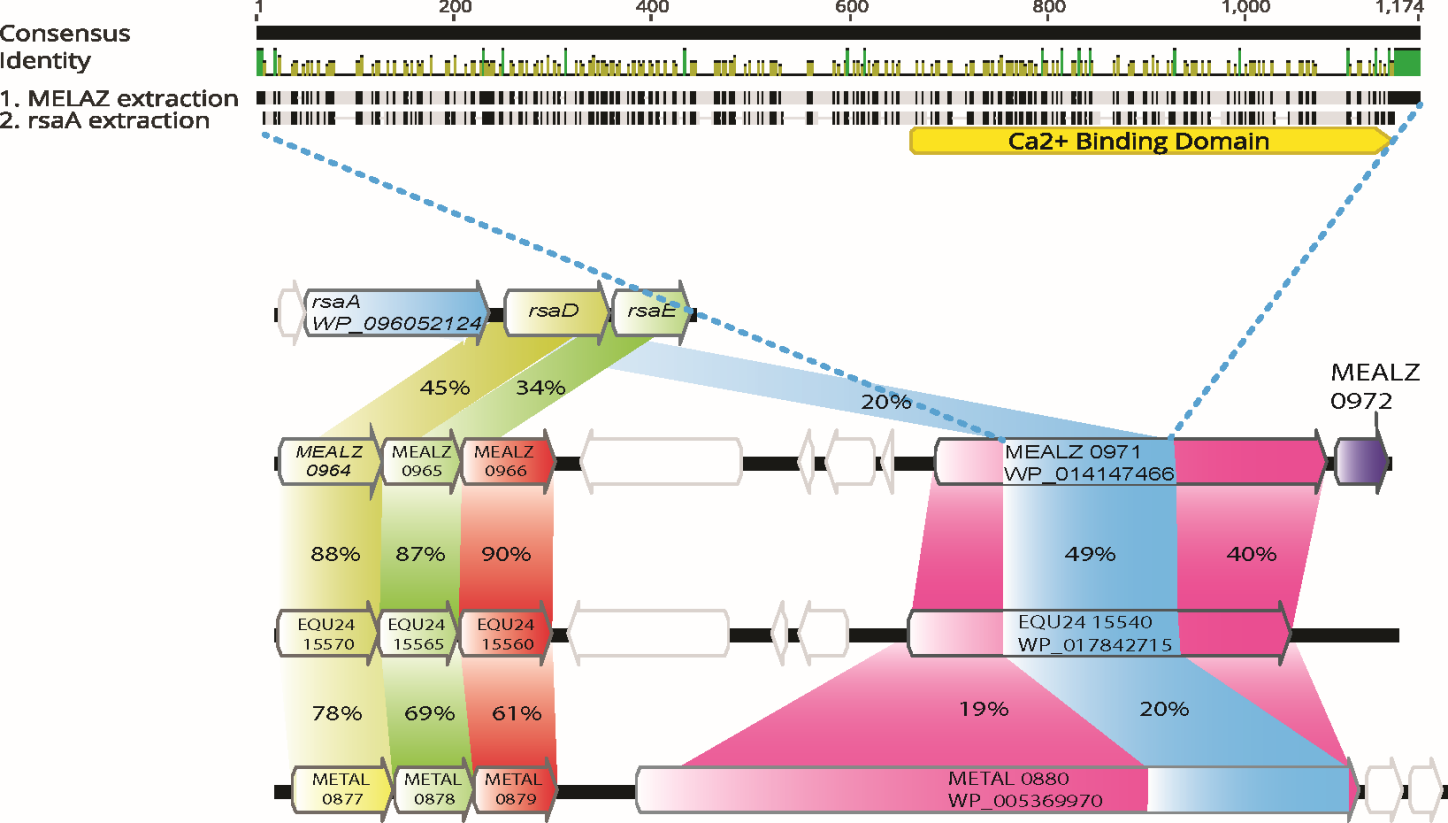
Comparison of identified gene loci in the genome and protein homology in *M. alcaliphilum 20Z^R^* and *M. buryatense 5* GB1 to known SLP protein (RsaA) in *C. crescentus* and identified homolog in *M. album BG8*. The homology of RsaA to each predicted SLP protein is highlighted in blue. RsaD and RsaE proteins for T1SS were compared for similarity, highlighted in yellow and green, respectively. *C. crescentus* is lacking the putative type 1 secretion outer membrane protein (OMP),which was compared for similarity in 20Z^R^, 5GB1, and BG8, highlighted in red. Lastly MEALZ_0972 highlighted in purple was solely identified in *M. alcaliphilum* 20Z^R^ and was in the same operon as the predicted S-layer protein.

The predicted SLPs were compared to the well-characterized S-layer (RsaA) protein from *Caulobacter crescentus*. Assessment of the translated amino acid sequence of the predicted methanotrophic SLPs using the multiple alignment program MAFFT showed that they have 20.6% AA sequence identity to RsaA (Fig. 2). The *rsaA* gene lies in the same operon as two of the three type 1 secretion system (TISS) genes, permease/ATPase (*rsaD*) and the periplasmic adapter subunit (*rsaE*). Upstream of both proposed S-layer genes in 20Z^R^ and 5GB1 lie all three genes for the TISS, a common arrangement for S-layer genes, with the permease/ATPase of *M. alcaliphilum* 20Z^R^ having 45% identity and the periplasmic adapter subunit having 34% identity with *Caulobacter crescentus* (Fig. 2) (26, 27). The calcium-binding domain of the RsaA protein from *Caulobacter* lays in homologous regions of the predicted S-layer proteins (7, 28). Additional homologs for the S-layer protein of *M. alcaliphilum* 20Z^R^ were identified using BLAST in the genomes of other methanotrophs known to have S-layers: *Methylomicrobium album* BG8 (30% AA sequence identity using BLAST and 19% AA sequence identity using MAFFT) and *Methylosarcina lacus* (33% AA sequence identity), as well as in other halophiles or haloalkaliphiles, such as *Halomonas salina* (48% AA sequence identity), *Desulfonatronum thioautotrophicum* (38% AA sequence identity), *Desulfonaspira thiodismutans* (33% AA sequence identity), *Oceanicola* sp. HL-35 (33% AA sequence identity), and *Marinobacterium litorale* (29% AA sequence identity). The phylogenetic analysis revealed that the SLP proteins *M. alcaliphilum* 20Z^R^ and *M. buryatense* 5GB1 cluster with proteins from two other methanotrophs*—Methylosarcina lacus* and *Methylomicrobium album* BG8, SLP proteins from *Deinococcus ratiodurans* and *Geobacter stearothermophilus*, as well as archaeal strains *Nitrosopumilus maritimus*, *Haloferax volcanil,* and *Sulfolobus* sp*.* (Fig. S1).

Overall, the homology of the MEALZ_0971 protein with other proteins from bacteria known to have S-layers, as well as characterized SLP proteins and its location adjacent to predicted T1SS, suggested the protein and associated small protein MEALZ_0972 can be the S-layer proteins.

### Protein structure prediction: Alphafold

**Fig 3:**
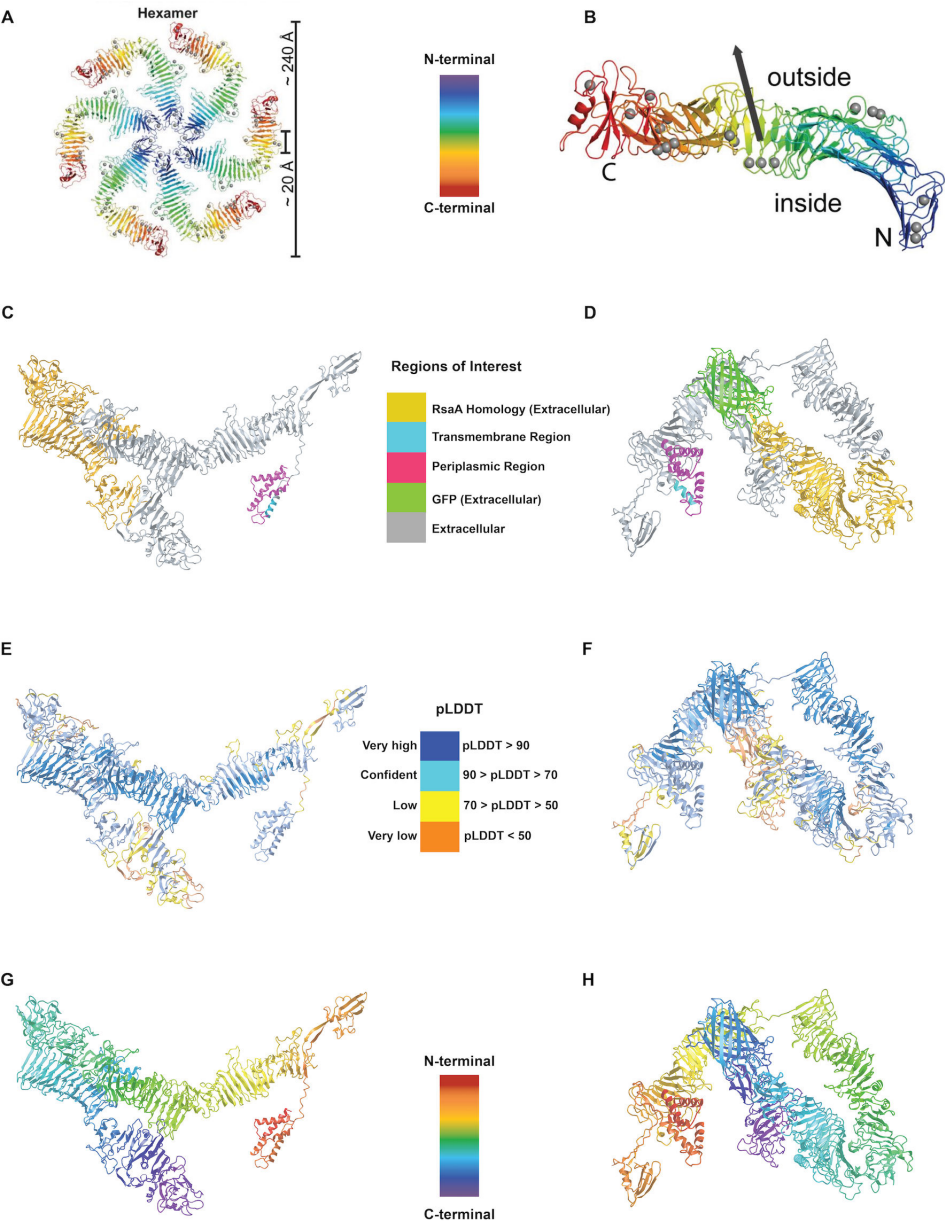
AlphaFold predictions of the putative SLP protein from *M. alcaliphilum* 20Z^R^ (protein MEALZ_0971) in comparison to the RsaA protein. A–B. RsaA structure, adapted from Bharat, T.A., *et al*. 2017. A: RsaA oriented in hexamer structure (PDB Code:5N97). B: RsaA monomer. Blue to red colors indicate the order of amino acid residues of the RsaA protein from N-terminal to C-terminal regions, respectively; The dark blue region represents the LPS-binding domain of the RSA protein (PDB Code:6Z7P). C–D. AlphaFold prediction of the MEALZ_0971, a putative SLP protein structure (**C**) and the structure of the MEALZ_0971-sfGFP protein fusion. The gold regions represent the SLP protein region homologous to RsaA. The predicted transmembrane region of the MEALZ_0971 protein is marked in blue. The putative periplasmic region of the protein is marked in pink. The light green represents the sfGFP structure. E. Confidence scores from high (blue) to low (orange) for the AlphaFold prediction of the MEALZ_0971 protein structure. F. Confidence scores from high (blue) to low (orange) for the AlphaFold prediction of the MEALZ_0971–sfGFP fusion structure. G–H. Predicted structure of the MEALZ_0971 (**G**) and MEALZ_0971–sfGFP fusion proteins. N-terminal and C-terminal residues are marked in red and purple, respectively.

S-layer proteins are typically attached to lipopolysaccharides (LPS) by noncovalent interactions in most studied Gram-negative bacteria (7, 26, 29). To characterize the MEALZ_0971 protein cell attachment, we compared it to the already characterized cryo-EM S-layer protein RsaA from *Caulobacter crescentus* (7). The N-terminal residues (up to 186) of the RsaA protein contain the membrane-proximal LPS-associated domain (7). The rest (residues 249–1,026) of the protein assembles in hexamers with N-terminal regions localized next to each other to form the outer-membrane lattice (Fig. 3A). The MEALZ_0971 protein homology to the RsaA protein is located toward the C-terminal end (Rsa_234-973_, highlighted in gold in Fig. 3C). Thus, the homology region does not include the LPS-binding portion of the RsaA protein, nor the C-terminal region, which is predicted to be critical for its protein secretion. DeepTMHMM investigation of the MEALZ_0971 sequence predicted a transmembrane region positioned at the N-terminal (residues 86–95, highlighted in purple, Fig. 3C). While the analyses suggest that the protein might also be anchoring to the membrane by its N-terminal region, the mechanism of attachment differs from the RsaA. Several attachment strategies are described for S-layer proteins, including posttranslational modifications, such as lipidation, transmembrane or membrane-binding domains, and interactions with other membrane proteins (30, 31). Considering the presence of the transmembrane domain as well the co-expression of the protein with MEALZ_0972, we suggest a possibility of a complex anchoring machinery. Further studies are needed to resolve the mechanistic aspects of the putative S-layer protein from *M. alcaliphilum* 20Z^R^, such as folding, transport, assembly, and attachment.

### Mutagenesis and scanning electron microscopy (SEM) studies

To determine if the identified proteins were responsible for the formation of the S-layer, the corresponding genes were subjected to mutagenesis. Single MEALZ_0971 (20Z^R^∆0971) and MEALZ_0972 (20Z^R^∆0972) knockout mutants of *M. alcaliphilum* 20Z^R^ were constructed. Single knockouts of *M. buryatense* 5GB1 genes EQU24_15540 (5GB1∆15540) and EQU24_07680 (5GB1∆*corA*) as well as complemented mutants of 5GB1∆15540 (5GB1∆15540::P89 EQU24_15540) were generated and provided for this study by Dr. M. E. Lidstrom (University of Washington). The 5GB1∆*corA* mutant was included to investigate the protein’s role in S-layer assembly.

**Fig 4:**
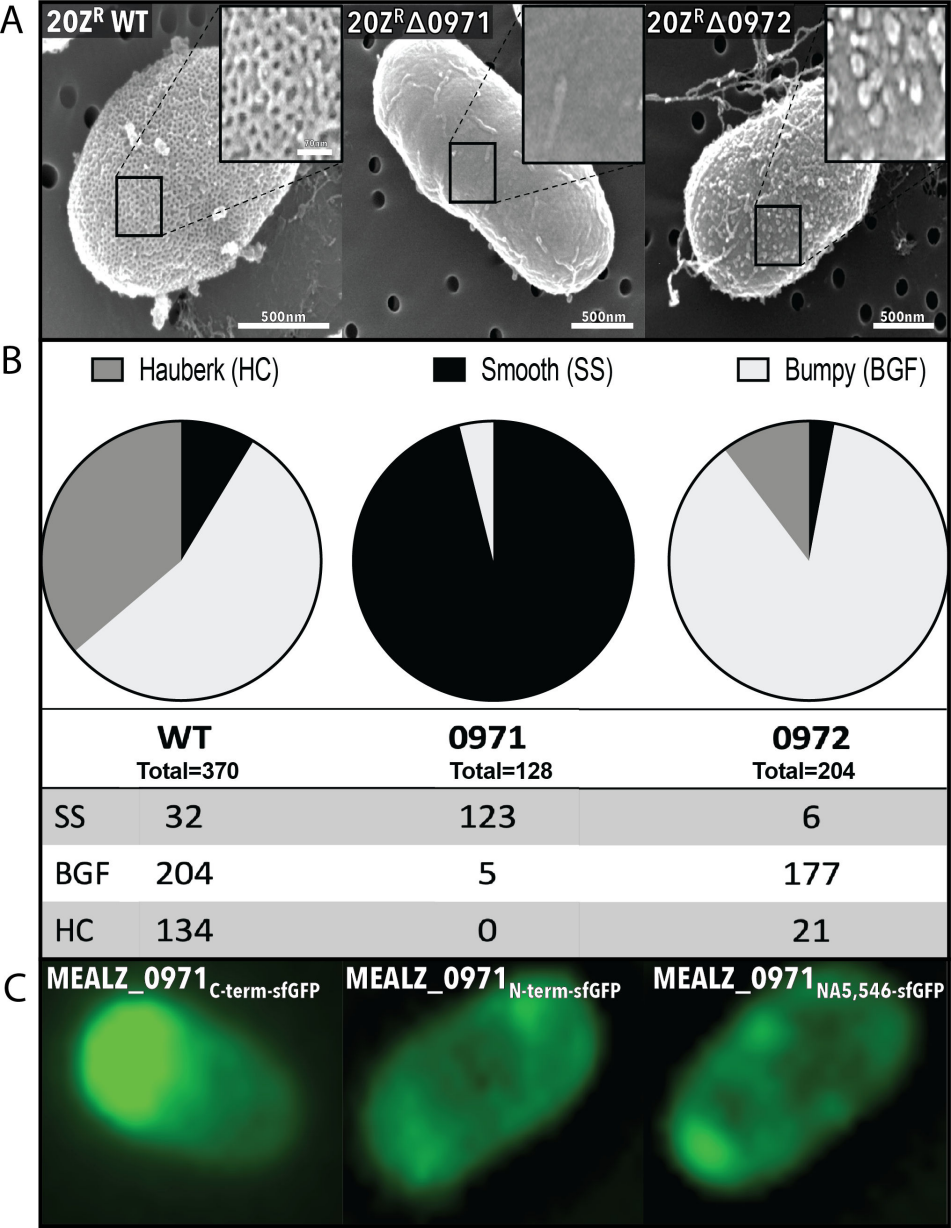
Analysis of phenotypic changes of the surface of the cells with proposed surface layer protein mutants using both scanning electron microscopy (SEM) and fluorescent microscopy. A: SEM of the SLP on 20Z^R^ WT cells, 20Z^R^ with MEALZ_0971 knocked out, and 20Z^R^ with MEALZ_0972 knocked out. B: Quantification using SEM micrographs of the surface of *M. alcaliphilum 20Z^R^*, *M. alcaliphilum 20Z^R^*∆0971, and *M. alcaliphilum 20Z^R^*∆0972. Surface represented as displaying Hauberk crystallized SLP, bumpy unorganized SLP, or smooth cell surface. C: Fluorescence microscopy of sfGFP integrated into the SLP at the C-terminal, N-terminal, and at the NA5,546 position from the start codon.

All mutants and the wild-type cultures were analyzed by scanning electron microscopy (SEM) (see *Materials and Methods*) to verify any changes in the morphology of the cell envelope, specifically the hexagonal matrix. As expected, the scanning electron micrographs of WT cells of *M. alcaliphilum* 20Z^R^ (Fig. 4A) and *M. buryatense* 5GB1 (Fig. S2A) verified the S-layer phenotype is present as arrays of hexagonal pits (i.e., the hauberk envelope). The pore-to-pore distance of the hauberk envelope in *M. alcaliphilum* 20Z^R^ is 333 ± 36 angstrom (Å). This is larger than pore-to-pore distances observed for the SLP protein lattices from *Caulobacter crescentus* (220 Å), *Deinococcus radiodurans* (8186 Å), *Geobacillys stearothermophilus* (70–120 Å), *Sulfolobus* (128–211 Å), *Nitrosopumilus* (220 Å), and *Haloferax volcanil* (259 Å) (7, 32–36).

In contrast, the cell surface of the *M. alcaliphilum* 20Z^R^∆0971 and *M. buryatense* 5GB1∆15540 mutants displayed a morphology that is characteristic of the outer membrane, with smooth ridges and folds (Fig. 4A; Fig. S2B). The 20Z^R^∆0972 mutant had some hexagonal pits along the surface but also showed globular structures (i.e., bumps) all over the surface, unlike that of the 20Z^R^∆0971 mutant, which was mostly smooth (Fig. 4A). Cells of the 5 GB∆*corA* mutant cells, as well as complemented mutant 5GB1∆15540::P89 EQU24_15540, have a cell-surface morphology similar to that of the WT strain, indicating that the protein is not responsible for the formation of hauberk-like structures (Fig. S2C). These results suggest that MEALZ_0971 in *M. alcaliphilum* 20Z^R^ and EQU24_15540 in *M. buryatense* 5GB1 are the SLP proteins or somehow contribute to the assembly of the cell SLP-envelopes.

We also found that capturing micrographs of individual cells using electron microscopy can be challenging. The hauberk layer can be masked by the presence of glycosylation, the fixation protocol, and/or cell growth stage. Since we observed some smooth-surface cells even among WT cells, a qualitative assessment of cell morphology was conducted, and cells were divided into three main categories: hauberk-covered (HC), bumpy globular features (BGF), or smooth surface (SS). The *M. alcaliphilum* 20Z^R^ WT presented a total of 134 HC cells, 204 BGF cells, and 32 SS cells (Fig. 4B). The number of cells covered by the hauberk-like envelope was significantly reduced in *M. alcaliphilum* 20Z^R^∆0972, which presented a total of 21 HC cells, 177 BGF cells, and six SS cells (Fig. 4B). We were not able to find any HC cells in the *M. alcaliphilum* 20Z^R^∆0971 mutant. The cell population of the mutant was represented mostly by SS cells (123) and a few BGF cells (Fig. 4B).

### Superfolder green fluorescent protein (sfGFP) tagging and fluorescence microscopy

To further confirm the MEALZ_0971 protein localization, we constructed a set of translational fusions with sfGFP. The fluorescent marker was integrated into MEALZ_0971 of *M. alcaliphilum* 20Z^R^ at the N-terminal, C-terminal, and inside of the protein near the C-terminal region at the NA5546 position from a putative start codon of the protein. The AlphaFold-predicted protein demonstrated no change in configuration with sfGFP integrated at the sites (Fig. 3D show internal integration). All three strains were investigated using fluorescence microscopy. When the protein was tagged at the N-terminal (N_ter_), there was little accumulation in the cell; the protein was distributed evenly across the cell surface (Fig. 4C). When MEALZ_0971 was sfGFP-tagged internally at the NA5546 site, there was also little accumulation of the fluorescence signal within the cell (Fig. 4C; Fig. S3). Thus, the live-imaging data for the MEALZ_0971_Nter-sfGFP_ and internal MEALZ_0971_NA5546- sfGFP_ integration suggest that the MEALZ_0971 protein is associated with the *M. alcaliphilum* cell envelope. However, when MEALZ_0971 was tagged at the C-terminal end of the protein, there was an accumulation of sfGFP at the poles of the cell (Fig. 4C; Fig. S4). This may indicate impaired secretion of the protein caused by GFP integration at the C-terminal (C_term_).

### Phenotyping studies

**Fig 5:**
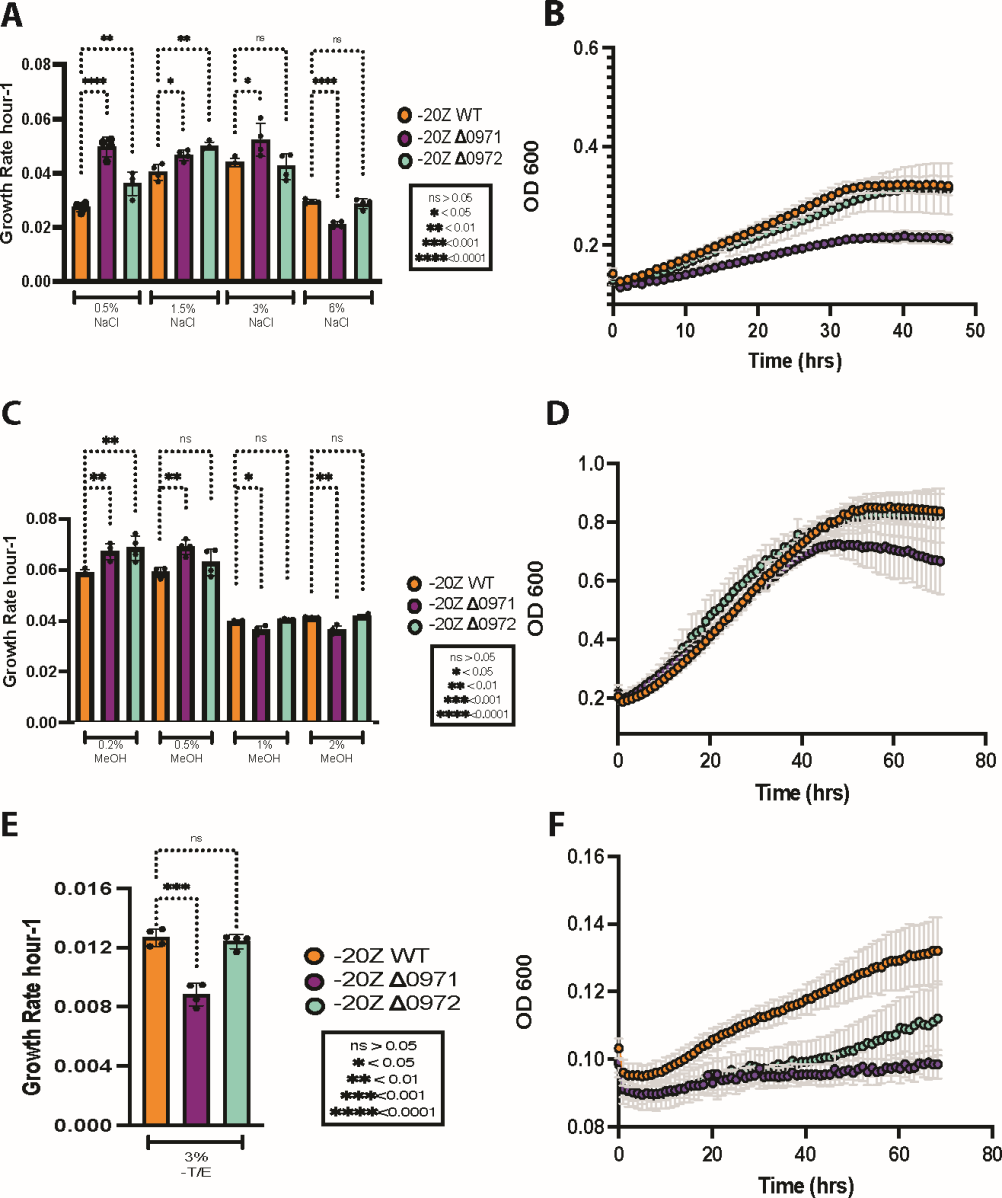
Growth rate of *M. alcaliphilum* 20Z^R^ and ∆MEALZ_0971 and ∆MEALZ_0972 mutants. A. Growth rates at different salinity 0.09–1.02 M (0.5%–6% NaCl wt/vol). B: Growth curve of 20Z^R^ WT, 20Z^R^ ∆0971, and 20Z^R^ ∆0972 grown with 6% NaCl. C: Growth rates at different methanol supplementation (0.2%–2% vol/vol). D: Growth curve of 20Z^R^ WT, 20Z^R^ ∆0971, and 20Z^R^ ∆0972 grown in 2% MeOH. E: Growth rates with trace element additions (T/E). F: Growth curve of 20Z^R^ WT, 20Z^R^ ∆0971, and 20Z^R^ ∆0972 grown without T/E. Unpaired *t*-test was used for statistical analysis of the growth rates of mutant strains compared to 20Z^R^.

Given the homology observed in S-layer proteins among halophilic microbes, subsequent testing was conducted to determine their potential role as an adaptation to the (hyper)saline environments. The WT and mutant strains were grown at low (0.5% NaCl), optimal (1.5%–3% NaCl), and elevated (6% NaCl) salinities (see *Materials and Methods*). At the optimal and low-salinity growth conditions, *M. alcaliphilum* 20Z^R^ ∆0971 had a higher growth rate (0.052±0.006 h^−1^) than WT and the ∆0972 mutant (Fig. 5A). However, when the strains were grown at the highest salinity of 6% NaCl, only the *M. alcaliphilum* 20Z^R^ ∆0971 strain showed a statistically significant decrease in the growth rate, which decreased from 0.030±0.001 to 0.021±0.001 h^−1^ (Fig. 5A and B). The data suggest that the MEALZ_0971 protein somehow improves the cell growth at high salinity. For further exploration of the phenotype, we investigated two cellular process that might be impacted by high salinity—envelop stability and metal uptake.

To further evaluate the MEALZ_0971 and MEALZ_0972 protein contribution to cell-envelope stability, the WT and mutant strains were grown in continually increasing methanol conditions from 0.2% to 2%. The mutant strains of *M. alcaliphilum* 20Z^R^ ∆0971 and ∆0972 showed higher growth rates compared to WT at lower methanol concentrations of 0.2% and 0.5% (Fig. 5C). When the methanol concentration was increased to 1%, 20Z^R^ ∆0971 had a significant decrease in growth rate from 0.040±0.001 to 0.036±0.001 h^−1^. When grown in 2% methanol, there was once again a significant decrease in growth rate from 0.041±0.001 to 0.036±0.001 (Fig. 5C and D). In contrast, the *M. alcaliphilum* 20Z^R^∆0972 mutant growth rate did not change significantly and was similar to that of the wild type. Together with the salinity tests, the data suggest that the surface layer protein contributes to cell envelope stability.

Finally, to evaluate the contribution of the S-layer proteins in metal uptake, the wild-type and mutant strains of *M. alcaliphilum* were grown under metal-limited conditions. All tests were carried out with the optimal salinity 0.51M (3% NaCl) with 0.2% methanol. However, the growth media was supplemented only with nitrogen (KNO_3_), sulfur (MgSO_4_), and phosphorus sources. When grown in medium lacking minerals, the 20Z^R^ ∆0971 showed a significant decrease in growth rate from the WT strain from 0.012±0.001 to 0.008±0.001 h^−1^, while the growth of 20Z^R^ ∆0972 mutant was similar to that of the WT (Fig. 5E and F).

Similar experiments were carried out with the *M. buryatense* 5GB1 WT and two mutants (5GB1∆*corA* and 5GB1∆15540). Under the mineral limitation, the mutant 5GB1∆*corA* showed a significant decrease in growth rates compared to 5GB1-WT, while the 5GB1∆15540 showed no difference (Fig. S5).

### Metal uptake: Inductively coupled plasma optical emission spectrometry (ICP-OES) data

To further test the role of S-layer proteins in metal uptake, the metal compositions of the cell biomass grown at optimal or metal-limited conditions were investigated (Table 1). At optimal conditions, the mutant cells showed higher uptake of Fe and Mn after normalization to account for varieties in biomass, while the rest were at the levels similar to those of the WT cultures.

**TABLE 1:** ICP-OES analysis of *M. alcaliphilum* 20ZR WT and mutant cells

Element			20Z^R^ + Cu			20Z^R^ Δ0971 + Cu			20Z^R^ Δ0972 + Cu			20Z^R^ - Cu			20Z^R^ Δ0971 - Cu			20Z^R^ δ0972 - Cu		
Nanomoles / g DCW	Transition metals	Mn	20	±	2	45	±	15	18	±	2	2	±	1	2	±	1	2	±	0
		Fe	6768	±	916	10623	±	2951	6401	±	674	1835	±	508	1908	±	239	1955	±	71
		Co	14	±	1	17	±	3	15	±	1	11	±	1	12	±	1	17	±	7
		Ni	16	±	3	22	±	6	22	±	3	15	±	1	15	±	6	12	±	2
		Cu	7412	±	421	7629	±	198	8007	±	601	344	±	8	369	±	17	429	±	180
		Zn	317	±	3	384	±	14	345	±	19	289	±	9	367	±	12	334	±	28
		Mo	6	±	1	10	±	1	7	±	1	4	±	0	4	±	1	6	±	1
		W	98	±	18	104	±	13	132	±	13	87	±	48	116	±	23	96	±	47
	Other biorelevant elements	Na	1418143	±	16478	1266003	±	70217	1582807	±	47876	1245372	±	153455	962106	±	111956	1504349	±	182316
		Mg	34852	±	946	38873	±	1142	39581	±	1718	27452	±	237	29812	±	151	38493	±	1180
		P	350069	±	3303	395670	±	10770	396969	±	13148	295296	±	5371	317555	±	8392	403333	±	4678
		K	749334	±	37216	800785	±	7802	751336	±	56780	749720	±	26004	708363	±	39609	966875	±	17388
		S	112490	±	1969	125496	±	2781	129801	±	3778	88927	±	2232	96139	±	2688	119752	±	1938
		Ca	2484	±	292	2783	±	341	2668	±	243	2122	±	116	2165	±	54	2811	±	167

## DISCUSSION

Many species of methanotrophic bacteria form a layer of self-assembled glycosylated proteins on the cell surface. However, the composition and function of additional cell envelope proteins have remained unsolved. This study was designed to identify the genetics of the S-layer matrix in gammaproteobacterial methanotrophs. Previous attempts to identify the matrix using targeted proteomics after polyacrylamide gel electrophoresis (PAGE) failed to detect the core protein. It has been speculated that once assembled, the S-layer matrix does not dissociate even after prolonged heat treatment in the presence of common detergents, like SDS, and reagents such as DTT or beta-mercaptoethanol (21). In this study, we used gene expression and nontargeted proteomics data to identify the most highly expressed genes in two methanotrophic bacteria. The initial hypotheses were as follows: (1) since the proteins form an envelope wrapped around the cell, they must be highly abundant and represented by a large protein; and (2) since the extracellular proteins are the most likely target for predator attachment or viral infection, they are under a strong selection pressure and should show little conservation among homologs from two closely related strains: 5GB1 and 20Z^R^ (37–39). We identified two proteins that fit all criteria—the MEALZ_0971 gene in *M. alcaliphilum* 20Z^R^ and the EQU24_15540 gene in *M. buryatense* 5GB1. The genes are highly expressed and share 40% amino acid identity.

The comparative genomic studies showed that the proteins encoded by MEALZ_0971 and EQU24_15540 genes share some homology (up to 20%) with well-characterized surface layer protein (RsaA) in *C. crescentus*. The SEM images demonstrate that the deletion of MEALZ_0971 and EQU24_15540 leads to elimination of hauberk structures from the surface of the cells, indicating that the proteins contribute to S-matrix formation. The fluorescence microscopy imaging of two cellular populations with translational sfGFP fusions at the N_ter_ and mid-position of the MEALZ_0971 protein showed its cell-surface localization. The Z-stack images suggest that the protein is forming an envelope that coats the cells. Based on these data, we suggest that MEALZ_0971 and EQU24_15540 encode alkaliphilic methanotroph S-layer core proteins.

One of the translational fusions, MEALZ_0971_Cter-sfGFP_ provided initial insights into the secretion mechanism. When MEALZ_0971 was tagged at the C-terminal loci, secretion was disrupted, supporting the idea that it is being secreted by the T1SS since proteins transported through T1SS have C-terminal recognition sites (40). The T1SS secretion genes are part of the *rsaA-*operon in *C. crescentus*, and the system has been shown to secrete the S-layer protein (5). Gene homologs of all three components of T1SS secretion were found upstream of MEALZ_0971 and EQU24_15540. It is tempting to speculate that the genes encode the S-layer protein secretion machinery; however, this needs to be validated by additional studies.

Once the genetics of the S-layers were identified, the next task was to gain insights into functional significance in methanotrophs. Our data showed that MEALZ_0971 and possibly MEALZ_0972 proteins stabilize the cell envelope at high salinities and high methanol conditions. Furthermore, the mutants displayed reduced growth in metal-limited environments, suggesting a role in metal uptake. To further investigate the role of the MEALZ_0971 and MEALZ_0972 proteins in metal uptake, we investigated the metal content of wild-type and mutant cell biomass through ICP-OES. No significant changes were observed, except for somewhat higher accumulation of iron and manganese in the mutants.

The deletion of *corA*, that encodes a putative copper-binding and S-layer-associated protein, did not impact the assembly of hauberk-like structures on the cell surface in *M. buryatense* 5GB1. Furthermore, the *M. buryatense* 5GB1∆*corA* grew normally on methane (data not shown). The 5GB1∆*corA* phenotype contradicted the phenotypes of *M. alcaliphilum* 20Z^R^∆*corA* and *M. album* BG8 ∆*corA*, which lost the ability to grow in methane and are weakly associated with S-layers with the cell surface (17, 18). The -omics data strongly suggest that CorA and CorB are regulated by copper availability. However, the lack of this copper-binding enzyme does not impact the growth of *M. buryatense* 5GB1 in the same way as it does to *M. alcaliphilum* 20Z^R^, most likely because contrary to 20Z^R^, the 5GB1 strain also possesses the iron-dependent soluble methane monooxygenase system (sMMO) and, thus, it can grow without copper under methane growth conditions (41).

We also found a few intriguing correlations—the growth phenotype of the 5GB1∆15540 followed the phenotype of the CorA mutant. The CorA protein has been suggested to contribute to copper uptake. We suggest that the SLP scaffold acts as a dock for other proteins involved in metal uptake. Additional studies are underway to confirm this function of the S-layer matrix.

Overall, it appears that methanotrophic SLPs do not significantly impact cell growth under optimal growth conditions; however, they become more essential under different types of membrane stability stressors. Considering that membrane stability and transport functions are interconnected, further investigations are needed to better understand the contribution of SLPs to metal transport.

## MATERIALS AND METHODS

### Strains and growth conditions

*M. buryatense* 5GB1C and *M. alcaliphilum* 20Z^R^ strains were grown at 30°C with methane (25% headspace) or 0.2% methanol on a nitrate mineral salt medium with 0.13M (0.75%) NaCl for 5GB1 and 0.51M (3%) NaCl for 20Z^R^ at pH 9 (42). For all 20Z^R^ phenotyping studies, the strains were grown in 48-well plates in a SpectraMax iD5 Multi-Mode Microplate Reader. Cultures were first grown in the specified medium in a 48-well plate until they reached exponential growth around an optical density of 0.3–0.6. These cultures were then used to inoculate the microplate wells with fresh media to an initial OD of 0.1 (total volume 400 µL). The setting in the plate reader was kept at a temperature of 30°C with continuous shaking on high in orbital motions. Optical density readings were taken every hour for 3 days or until cultures reached the stationary phase of growth. For 5GB1 phenotyping studies, the strains were grown in 125-mL shake flasks (with 25 mL media). Cultures were first grown in the specified medium till they reached exponential growth of 0.3–0.6 and inoculated into fresh medium to an initial OD of 0.1 (total culture volume 25 mL).

The following *Escherichia coli* strains were used for cloning and gene transfer experiments: *E.coli* S17-1 (lab stock), *E.coli* DH5α (New England Biolabs (NEBlab), Catalog Number C2987H, *fhuA2Δ(argF-lacZ)U169 phoA glnV44 Φ80Δ(lacZ)M15 gyrA96 recA1 relA1 endA1 thi-1 hsdR17*) and *E. coli* DH10B (NEBlabs, Catalog Number C3019H: *Δ(ara-leu) 7697 araD139 fhuA ΔlacX74 galK16 galE15 e14- ϕ80dlacZΔM15 recA1 relA1 endA1 nupG rpsL (Str^R^) rph spoT1 Δ(mrr-hsdRMS-mcrBC*)). *E.coli* strains were grown using Luria–Bertani (LB) broth or LB agar media (Miller, Difco BD Life Sciences) and incubated at 37°C. The following antibiotics were applied when required: kanamycin (100 µg/mL) and rifamycin (50 µg/mL).

### Sequence alignment and comparison

The genomic sequences used for this study were *M. alcaliphilum* 20Z^R^ (FO082060.1), *M. buryatense* 5GB1 (CP035467.1), and *C. crescentus* (CP023315.3). The type 1 secretion system proteins and proposed S-layer proteins were compared to those of *C. crescentus* using the MAFFT online tool (43, 44). The outputs were then processed into Geneious to construct the comparative diagram (45).

### Alphafold and structural comparison methods

AlphaFold 2 (accessed with Tamarind Bio https://www.tamarind.bio/) was used to predict the protein structures of MEALZ_0971 and MEALZ_0972 with an insertion of superfolder green fluorescent protein (sfGFP) 1,000 bp upstream of the C-terminal end of the protein (46, 47). UniProt BLAST was used to find the RsaA homologous amino acid region in MEALZ_0971 (48). DeepTMHMM v1.0.24 was used to predict cellular locations (cytosol/periplasm, transmembrane, or extracellular) of amino acids in MEALZ_0971 (49). Protein Data Bank (PDB) files were visualized, and the predicted homologous RsaA, cytosolic/periplasmic, transmembrane, and extracellular amino acid regions were colored with Mol* from RCSB PDB (50).

### Transcriptomics expression comparison heatmap methods

A total of 59 RNA-seq raw reads from Akberdin *et al*., 2018, Vasquez, 2023, and unpublished studies from the C1-Lab at SDSU representing 27 different growth conditions varying in salinity, pH, nitrogen sources, carbon sources, and metalloenzyme cofactors such as copper (Cu), calcium (Ca), lanthanides (Lns), and tungsten (W) were used in this comparative gene expression study (23, 51). Raw RNA-seq reads were preprocessed with fastp (52). Transcript quantification was then performed with Salmon in the mapping-based mode with the validateMappings and automatic library type detection arguments against the transcriptome of *M. alcaliphilum* 20Z^R^ (GenBank Accession: FO082060.1 & FO082061.1) (53). The variance stabilizing transformation method from DESeq2 was used to normalize raw transcript counts (54). The gene expression heatmap was generated using the R pheatmap v1.0.21 package (55).

### Mutagenesis

The mutant strains of *M. buryatense*—5GB1∆*corA* (EQU24_07680) and 5GB1∆15540—were kindly provided by Dr. M.E. Lidstrom (University of Washington). The mutants of *M. alcaliphilum* 20Z^R^ were generated using conjugation with the pCM433 vector as described previously (23, 42, 56). The upstream and downstream flanking regions of the target gene were amplified by PCR using Thermo Scientific Phusion Flash PCR Master Mix. These flanking regions were ligated into the linear form of the pCM433 backbone using NEBuilder HiFi DNA Assembly Master Mix and transformed into NEB 10-beta competent *E. coli* cells. Colonies were tested by colony PCR as described (42). Positive constructs were then transformed into *E. coli* S17-1 cells used for bi-parental conjugation with *M. alcaliphilum* 20Z^R^. The strain then underwent through selection on kanamycin followed by counterselection with sucrose to knockout the vector. Colonies were then tested for knockout of each gene by PCR and validated by sequencing.

### Translational fusions of MEALZ_0971 with sfGFP

To tag MEALZ_0971 with superfolder green fluorescent protein (sfGFP), the pCM433 allelic exchange vector was used (42). The sfGFP was codon-optimized to 20Z^R^ based on its codon usage bias (CUB) table previously identified (57). Vectors were constructed with flanking regions of the N-terminal, C-terminal, or position 5,546 bps to insert sfGFP into each location of the MEALZ_0971. Positive mutants were identified, the tagged gene was amplified using PCR, and correct integration was confirmed by sequencing.

### Imaging studies: Scanning electron microscopy (SEM)

The imaging studies were carried out as previously described (58), with the following modification: a fixative solution (2.5% glutaraldehyde, 0.1 M cacodylate buffer (CB) was supplemented with 0.5% NaCl to maintain osmolarity during initial fixation. Samples were imaged at the SDSU Electron Microscopy Facility on an FEI Quanta FEG 450 at 20 kV accelerating voltage at a working distance of ~10 mm.

### Imaging studies: Fluorescence microscopy

Samples were prepared for fluorescence microscopy by growing cells to the exponential phase and collecting cells through centrifugation. The cells were then fixed in 4% formaldehyde for 1 hour and washed 3X with the NMS medium. Samples were mounted and images on an all-in-one Kenence fluorescent microscope BZ-X Series.

### ICP-OES analyses

Cell pellets (*n* = 3 for each strain) were collected via centrifugation. Each sample (about 20 mg dry cell weight, DCW) was digested in concentrated (68%–70%) ultrapure nitric acid at 65°C for 2–24 hours (until all solids were completely dissolved). Samples were then diluted to 5 mL with the addition of 4.5 mL of ultrapure distilled water. Analysis was performed using an Agilent 5800 ICP-OES with an SPS 4 Autosampler. Three replicates of 1.5 mL per sample were analyzed, and the averaged value was used to determine concentrations. Read time was set to 5 seconds, RF power was set to 1.4 kW, stabilization time was 15 seconds, the nebulizer flow was set to 0.70 L/min, the plasma flow 12.0 L/min, Aux flow at 1.00 L/min, and the make-up flow set to 0. Measurements were done using the axial viewing mode. Wavelengths were chosen to minimize spectral and chemical interference, and the Agilent software performed peak-fitting to minimize spectral interference. Where interference was high, multiple wavelengths were chosen and averaged to get the reported concentration.

### Phylogenetic analyses

The analyses were based on 16 amino acid sequences of the putative or validated SLP proteins, including *Methylotuvimicrobium alcaliphilum* 20Z^R^, *Methylotuvimicrobium buryatense* 5GB1, *Methylomicrobium album* BG8, *Caulobacter crescentus, Deinococcus radiodurans*, *Geobacillus stearothermophilus*, *Sulfolobus*, *Nitrosopumilus maritimus* SCM1, *Haloferax volcanii*, *Methylosarcina lacus*, *Halomonas salina*, *Desulfonatronum thioautotrophicum*, *Desulfonatronospira thiodismutans*, *Oceanicola* sp. HL-35, *Marinobacterium littorale*, and *Acidovorax sp*. KKS102. Multiple sequence alignments were performed on these sequences using MAFFT version 7.520 (59), utilizing the global pairwise alignment (1000 iterative refinements) strategy suitable for sequences with conserved regions. To improve the quality of the alignment for phylogenetic analysis, the initial alignment was trimmed using trimAl version 1.4.rev22 (60). Positions with gaps in more than 85% of the sequences were removed to exclude poorly aligned or ambiguously aligned regions. The trimming was performed with the following command: trimAl -in aligned_sequences.fasta -out trimmed_alignment.fasta -gt 0.85. A maximum likelihood phylogenetic tree was constructed using IQ-TREE version 2.3.6 (61). To assess the robustness of the inferred phylogenetic relationships, 1,000 ultrafast bootstrap replicates were computed to generate a consensus tree. *Oceanicola sp*. HL-35 was selected as an outgroup. The rooted tree was visualized using Interactive Tree Of Life (iTOL) version 6 (62).

## SUPPLEMENTAL MATERIAL

Figure S1 aem.01364-24-s0001.tiff Phylogenetic tree constructed using 16 amino acid sequences from characterized surface layer proteins and genes with homology to MEALZ_0971.

Figure S2 aem.01364-24-s0002.tiff Analysis of phenotypic changes of the surface of the 5GB1 cells with proposed surface layer proteins mutants using scanning electron microscopy.

Figure S3 aem.01364-24-s0003.mp4 Z-stack images of the M. alcaliphilum 20ZR strain expression MEALZ_0971 proteins tagged with sfGFP at the N-terminal region.

Figure S4 aem.01364-24-s0004.mp4 Z-stack images of the M. alcaliphilum 20ZR strain expression MEALZ_0971 proteins tagged with sfGFP at the C-terminal region.

Figure S5 aem.01364-24-s0005.tiff Characterization of growth dynamics in M. buryatense wild type and mutant strains 5GB1 ∆corA and 5GB1 under different growth conditions.

Supplemental legends aem.01364-24-s0006.docx Legends for Fig. S1 to S5.

ASM does not own the copyrights to Supplemental Material that may be linked to, or accessed through, an article. The authors have granted ASM a non-exclusive, world-wide license to publish the Supplemental Material files. Please contact the corresponding author directly for reuse.
