# Supplementary figures and images for: Microbial hauberks: composition and function of surface layer proteins in gammaproteobacterial methanotrophs
Source: Appl Environ Microbiol. 2024 Dec 31;91(1):e01364-24. doi: 10.1128/aem.01364-24 (PMC11784148; doi:10.1128/aem.01364-24)

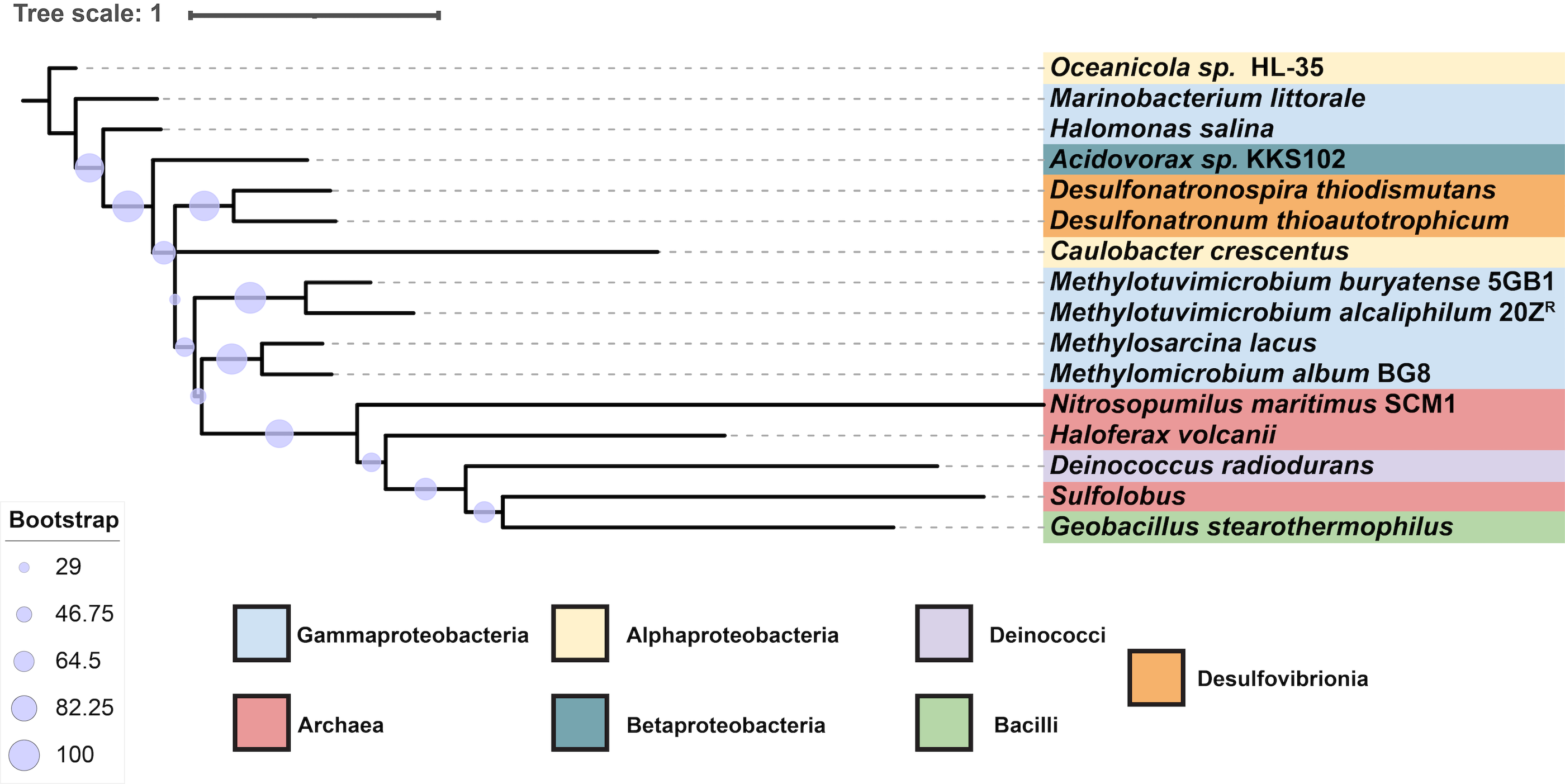

Supplement: Figure S1 — Phylogenetic tree constructed using 16 amino acid sequences from characterized surface layer proteins and genes with homology to MEALZ_0971. [file aem.01364-24-s0001.tiff]

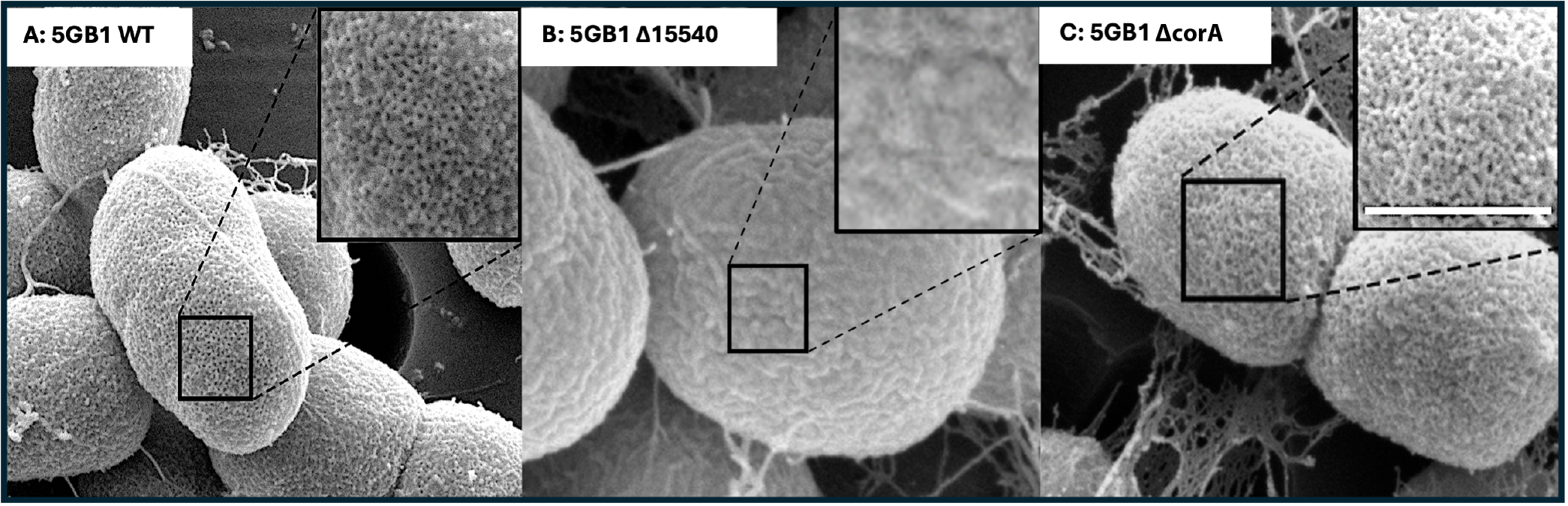

Supplement: Figure S2 — Analysis of phenotypic changes of the surface of the 5GB1 cells with proposed surface layer proteins mutants using scanning electron microscopy. [file aem.01364-24-s0002.tiff]

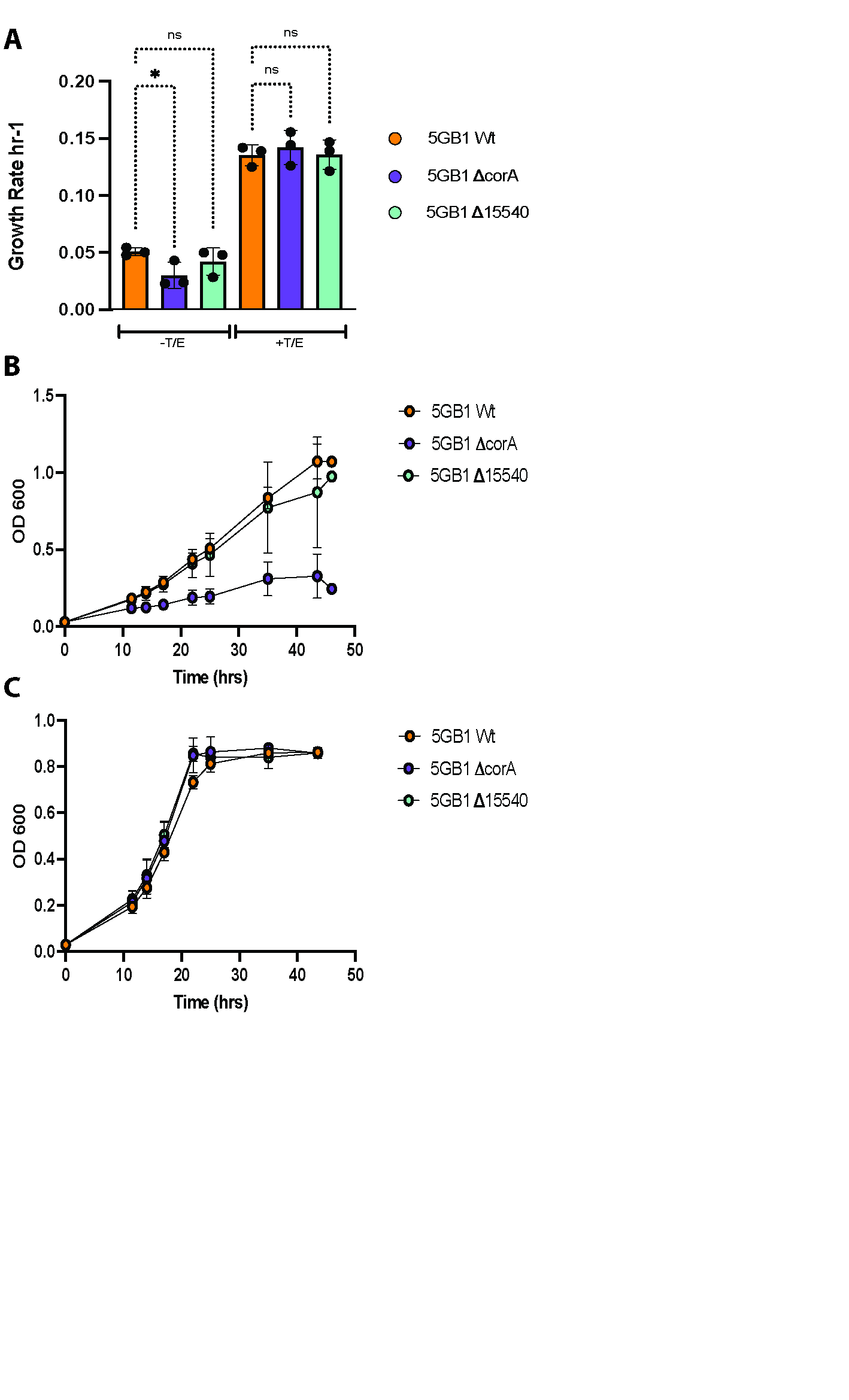

Supplement: Figure S5 — Characterization of growth dynamics in M. buryatense wild type and mutant strains 5GB1 ∆corA and 5GB1 under different growth conditions. [file aem.01364-24-s0005.tiff]
