# Supplementary material for: Microbial hauberks: composition and function of surface layer proteins in gammaproteobacterial methanotrophs
Source: Appl Environ Microbiol. 2024 Dec 31;91(1):e01364-24. doi: 10.1128/aem.01364-24 (PMC11784148; doi:10.1128/aem.01364-24)
Supplement: Supplemental legends — Legends for Fig. S1 to S5. [file aem.01364-24-s0006.docx]

**RE:** *Microbial Hauberks: Composition and Function of Surface Layer Proteins in Gammaproteobacterial Methanotrophs*. By Richard Hamilton, William Gebbie, Chynna Bowman, Alex Mantanona, and Marina G. Kalyuzhnaya.

**Supplementary Figures**

**Figure S1.** Phylogenetic tree constructed using 16 amino acid sequences from characterized

surface layer proteins and genes with homology to MEALZ_0971.

**Figure S2.** Analysis of phenotypic changes of the surface of the 5GB1 cells with proposed surface layer proteins mutants using scanning electron microscopy (SEM) A: SEM of the surface of 5GB1 WT cells. B: SEM of the surface of 5GB1 with EQU24_15540 knocked out. C: SEM of the surface of 5GB1 with EQU24_07680 (*corA*) knocked out. SEM Micrographs provided from David Collins (Collins 2021).

**References**

Collins, D. A. (2021). Dissecting the cellular biology of the model methanotroph *Methylotuvimicrobium alcaliphilum* 20Z^R^ through multi-omics, mutagenesis, and microscopy, San Diego State University.

**Figure S3.** Z-Stack images of the *M.alcaliphilum* 20Z^R^ strains expression MEALZ_0971 proteins tagged with sfGFP at the N-terminal region. Cells were stained with FM4-64 membrane dye (red) and DAPI. (blue)

**Figure S4.** Z-Stack images of the *M.alcaliphilum* 20Z^R^ strains expression MEALZ_0971 proteins tagged with sfGFP at the C-terminal region. Cells were stained with FM4-64 membrane dye (red) and DAPI.

**Figure S5.** Characterization of growth dynamics in M.buryatense wild type and mutant sStrains 5GB1 ∆corA and 5GB1 under different growth conditions. A: Growth rates of 5GB1 WT, 5GB1∆corA, and 5GB1∆15540 grown in metal limited/ sufficient conditions. B: Growth curve of 5GB1 WT, 5GB1∆corA, and 5GB1∆15540 grown in metal limited conditions. C: Growth curve of 5GB1 WT, 5GB1∆corA, and 5GB1∆15540 grown in metal sufficient conditions limited conditions.
